# Environmental Status Assessment Using DNA Metabarcoding: Towards a Genetics Based Marine Biotic Index (gAMBI)

**DOI:** 10.1371/journal.pone.0090529

**Published:** 2014-03-06

**Authors:** Eva Aylagas, Ángel Borja, Naiara Rodríguez-Ezpeleta

**Affiliations:** AZTI-Tecnalia, Marine Research Division, Sukarrieta, Bizkaia, Spain; Swansea University, United Kingdom

## Abstract

Marine ecosystem protection and conservation initiatives rely on the assessment of ecological integrity and health status of marine environments. The AZTI's Marine Biotic Index (AMBI), which consists on using macroinvertebrate diversity as indicator of ecosystem health, is used worldwide for this purpose. Yet, this index requires taxonomic assignment of specimens, which typically involves a time and resource consuming visual identification of each sample. DNA barcoding or metabarcoding are potential harmonized, faster and cheaper alternatives for species identification, although the suitability of these methods for easing the implementation of the AMBI is yet to be evaluated. Here, we analyze the requirements for the implementation of a genetics based AMBI (gAMBI), and show, using available sequence data, that information about presence/absence of the most frequently occurring species provides accurate AMBI values. Our results set the basics for the implementation of the gAMBI, which has direct implications for a faster and cheaper marine monitoring and health status assessment.

## Introduction

Increasing human activities in seas and oceans are likely to produce impacts on marine ecosystems [1], [2]. Yet, the United Nations Convention on the Law of the Sea [3], further supported by the 1992 Convention on Biological Diversity [4], establishes an international obligation to sustainably use marine resources. Additionally, several national or regional initiatives (e.g. the Australian Oceans Policy, the Canadian Oceans Act and Oceans Strategy, the USA Oceans Act, and the European Water and Marine Strategy Framework Directives (WFD, 2000/60/EC and MSFD, 2008/56/EC)) have been developed to protect, conserve or enhance marine ecosystems. These initiatives rely on the assessment of ecological integrity and marine health status [5], which requires adequate and rigorous spatiotemporal monitoring of multiple ecosystem components [6]–[8].

Among the components to be monitored, marine benthic macroinvertebrates are frequently used as indicators of ecosystem health. Benthic indices summarize complex biological information such as community composition in a single number that ranks sites on a scale from good to bad status [9]. Numerous different benthic indices have been developed in recent times [10], [11], allowing managers to identify impacted sites and decide on habitat restoration measures. One of the most successful indices used worldwide is the AZTI's Marine Biotic Index (AMBI) [12], which is officially used in many European countries and has been tested in America, Africa, Asia and Oceania [13], where examples of its application can be found [9], [14].

AMBI is based on abundance-weighted pollution tolerances of the species present in a sample, with tolerance being expressed categorically as one of five ecological groups (sensitive to pressure, indifferent, tolerant, opportunist of second order and opportunist of first order). Currently a list of about 6,000 worldwide species with ecological group assigned is available (http://ambi.azti.es). In addition, Warwick et al. [15] and Muxika et al. [16] have proposed the use of this index based upon presence/absence and biomass of species (p/a AMBI and BAMBI, respectively). All forms of AMBI require each species to be sorted and identified under a stereomicroscope. This is a time and resource consuming process that has limitations in some cases, as for example when damaged specimens o immature life stages are present [9].

Despite the importance of monitoring and assessment, the current economic crisis is leading some countries to pay attention on their monitoring budgets [17]. This fact has led researchers to investigate new and cost-effective methods to monitor and assess marine waters [18]. Genomic methods are a promising avenue to analyze biological systems, especially due to the recent advent of high-throughput sequencing technologies [19]. Among these methods, DNA barcoding and metabarcoding have the potential to increase speed, accuracy and resolution of species identification, while decreasing its cost in biodiversity monitoring [20].

Barcoding consists of taxonomically assigning a specimen based on sequencing a short standardized DNA fragment (barcode). In the metabarcoding approach, the analysis is extended to a community of individuals (of different species) rather to a single individual [20], [21]. In both cases, sequences need to be compared to a reference library that contains the correspondence between the barcodes and taxonomical classification. Several studies have used “metabarcoding” to study marine and tropical rainforest meiofauna [22], soil fauna [23], arthropods [20], [24], zooplankton [25] and fish gut contents [26].

The efficiency and accuracy in taxonomic identification using metabarcoding largely depend on the targeted barcode, which should be taxonomically informative [27], and primer set used for amplification, which should be adequate for the target species [26]. Primers can therefore be group specific, if the goal is to describe the diversity of species of a specific taxonomic group (i.e. nematodes in sediments [22]), or wide range, if the goal is to obtain a comprehensive analysis of samples containing species from numerous phyla [26]. If required, a cocktail of wide range and group specific primers can be used to cover the comprehensive biodiversity of the samples under study [28].

For animals, the most commonly used barcode is a 658 bp section of the mitochondrial cytochrome c oxidase subunit 1 gene (*CO1*) [29]. This gene has a faster substitution rate, compared to nuclear rRNA genes, which makes it suitable for species discrimination [29]. Yet, alternatives have been developed for cases when *CO1* sequences are insufficient to distinguish recognized species [30] or when amplification is challenging [22]. Among the alternatives, the nuclear 18S small subunit rRNA (*18S rRNA*) is the most widely used [31], although other markers such as the nuclear *28S rRNA* and the mitochondrial *12S rRNA* have also been suggested [25], [32].

Attempting a (meta)barcoding approach for the AMBI calculation is challenging as the species that compose the index belong to different taxonomic groups. Searching the appropriate genetic markers and primers for the target organisms is mandatory to cover the maximum spectrum of species within a sample and therefore avoid underestimations. Furthermore, a large enough barcode reference library is needed to comprehensively determine the biodiversity in the samples. In the present study, we evaluate the potential of an AMBI based on taxonomic identification by (meta)barcoding. For that purpose, we analyze the genetic resources available for the AMBI species, and determine the minimum reference library size and content required to calculate an accurate index. Additionally, we identify the best primers to retrieve the most complete representation of the AMBI taxonomic diversity and provide sequences for 22 species for which no genetic resources were available.

## Methods

### Datasets: species, sequences and case studies

Species list and assignment into one of the five ecological groups defined by the index were retrieved from the AMBI 5.0 software (http://ambi.azti.es). Taxonomic classification of the 5,977 retrieved soft-bottom macroinvertebrate species was done through the World Register of Marine Species (WoRMS) (www.marinespecies.org) and verified in the European Register of Marine Species (ERMS) (www.marbef.org). Sequences of the mitochondrial cytochrome oxidase 1 (*CO1*) and nuclear 18S ribosomal RNA (*18S rRNA*) genes of the 5,977 species were searched in GenBank database (accession: July 2013) and retrieved when available. The case studies used for subsequent analyses consisted on a subset of 734 samples of soft-bottom macroinvertebrates collected during annual surveys conducted by the Littoral Water Quality Monitoring and Control Network of the Basque Country, northern Spain [33], in 32 and 51 coastal and estuarine stations between 1995 and 2001 and between 2002 and 2011, respectively. From the samples collected, 694 contain at least one individual and are the ones used in further analyses, being the remainder azoic.

### AMBI and p/a AMBI calculation and agreement measures

AMBI (calculated using the number of individuals of each species) and p/a AMBI (calculated using presence (p)/absence (a) of each species ignoring number of individuals) values were calculated based on the proportional occurrences of benthic macrofaunal species among five ecological groups according to the pollution gradient. This gradient ranges from Ecological Group I – species very sensitive to organic enrichment and present under unpolluted conditions, to Ecological Group V – first-order opportunistic species present in pronounced unbalanced situations, and is calculated using the formula: (p/a) AMBI = (0×% GI) + (1.5×% GII) + (3×% GIII) + (4.5×% GIV) + (6×% GV)/100, where percentages represent number of individuals (AMBI) or species (p/a AMBI) of each ecological group [12]. AMBI and p/a AMBI values are grouped in categorical pollution levels (i.e. quality classes): “unpolluted” from 0 to 1.2, “slightly polluted” from 1.3 to 3.3, “moderately polluted” from 3.4 to 5, “heavily polluted” from 5.1 to 6 and “extremely polluted” from 6.1 to 7. AMBI 5.0 software and an in-house R script were used for automated (p/a) AMBI value calculations.

Cohen's Kappa [34] was used to determine the agreement between pollution levels obtained for the same stations but using different species sets. The level of agreement is described using the ranges suggested by Monserud and Leemans for each value of Kappa [35]: <0.05, no agreement; 0.05–0.20, very poor; 0.20–0.40, poor; 0.40–0.55, fair; 0.55–0.70, good; 0.70–0.85, very good; 0.85–0.99, excellent and 1, perfect. In order to determine if the Kappa value obtained with the x most frequent species (x being 10, 25 and 50%) is significantly better than that obtained with the same number of species selected randomly, we subsampled 100 times x species and calculated the p/a AMBI of each station considering this subset of species. The Kappa of each of the 100 subsets was calculated with respect to the original species list and the confidence interval of the obtained distribution was used to assign a p value to the Kappa obtained with the most frequent species.

### Primer pair analysis

Primers designed to amplify *CO1* and *18S rRNA* gene fragments across representative species of marine macroinvertebrates were retrieved from the bibliography (Table S1 and Figure S1). From the total sequences for *CO1* and *18S rRNA* genes retrieved from GenBank, multiple sequences from the same species were removed by applying cd-hit [36] separately for each taxa. This program groups sequences according to a similarity threshold (which was set to 0.9 in this case) and selects the longest one as representative of the group.

Predicting the performance of a primer pair against a target sequence requires the putative annealing region of the primer to be present in the sequence. Because some of the retrieved sequences are partial and/or do not include the primer region, not all primer pairs can be tested against all sequences. Therefore, in order to avoid false negatives, we tested each primer pair only on the sequences that contain the putative annealing region. For that purpose we used the *CO1* region of the complete mitochondrial gene from *Mytilus galloprovincialis* (Accession number DQ399833) and the *18S rRNA* gene from *Aplysia punctata* (Accession number AJ224919) as reference to determine the most external nucleotide position of each primer for *CO1* and *18S rRNA* respectively. Then, each sequence was compared with the reference using BLAST [37] and, for each primer pair, only those included within the primer pair external positions were selected (See figure S1 for regions tested for each primer primer). Additionally, due to the low number of sequences to be tested for *CO1*, we retrieved a total of 3687 complete metazoan mitochondrial genome sequences (all those available) from the NCBI Organelle Genome Resources database (November 2013) (http://www.ncbi.nlm.nih.gov/genomes/OrganelleResource.cgi?taxid=33208), from where 84 sequences were selected for the analysis as belonged to species of the AMBI. Each primer pair was evaluated against its correspondent sequence set using PrimerProspector [38] with default parameters. For species that contained more than one sequence, if at least one of them amplifies, the species is considered positive for this primer.

### Animal samples, DNA extraction, PCR and sequencing

The stations that, according to the data series, contain the most frequent species were selected for DNA barcoding. For this purpose, specimens were manually separated, *visu* identified and preserved separately in ethanol until DNA extraction. Taxonomic identification was done by experts from the Cultural Society INSUB following the identification protocols accepted and applied by the scientific community. Total genomic DNA from 115 species belonging to 9 phyla (Annelida, Arthropoda, Cnidaria, Echinodermata, Mollusca, Nematoda, Nemertea, Plathyhelminthes and Sipuncula) was extracted from 1 mm^3^ of tissue (which in some cases, came from more than one individual) using the Wizard SV 96 Genomic DNA Purification System (Promega) following manufacturer's instructions. The 658 bp region of the *CO1* gene was amplified using the forward dgLCO-1490 and the reverse dgHCO-2198 degenerated primer pair [39]. All PCRs were performed in a 20 µl volume containing 1 X PCR buffer with 1.8 mM MgCl2, 3% DMSO, 0.2 mM dNTP, 1.25 U TAQ polymerase (ROCHE), 0.4 µM of each primer, and 80–100 ng of DNA template. The thermal cycling conditions were based on [39] and consisted of 95°C for 2 minutes; 35 cycles of 95°C for 40 seconds, 45°C for 40 seconds, and 72°C for 60 seconds, followed by a final extension of 72°C for 7 minutes and a final cooling at 4°C. PCR products were purified with ExoSAP-IT (AFFYMETRIX) and Sanger sequenced.

## Results and Discussion

### Species-level taxonomic identification but not species abundance is required for a reliable index calculation

**Figure 1:**
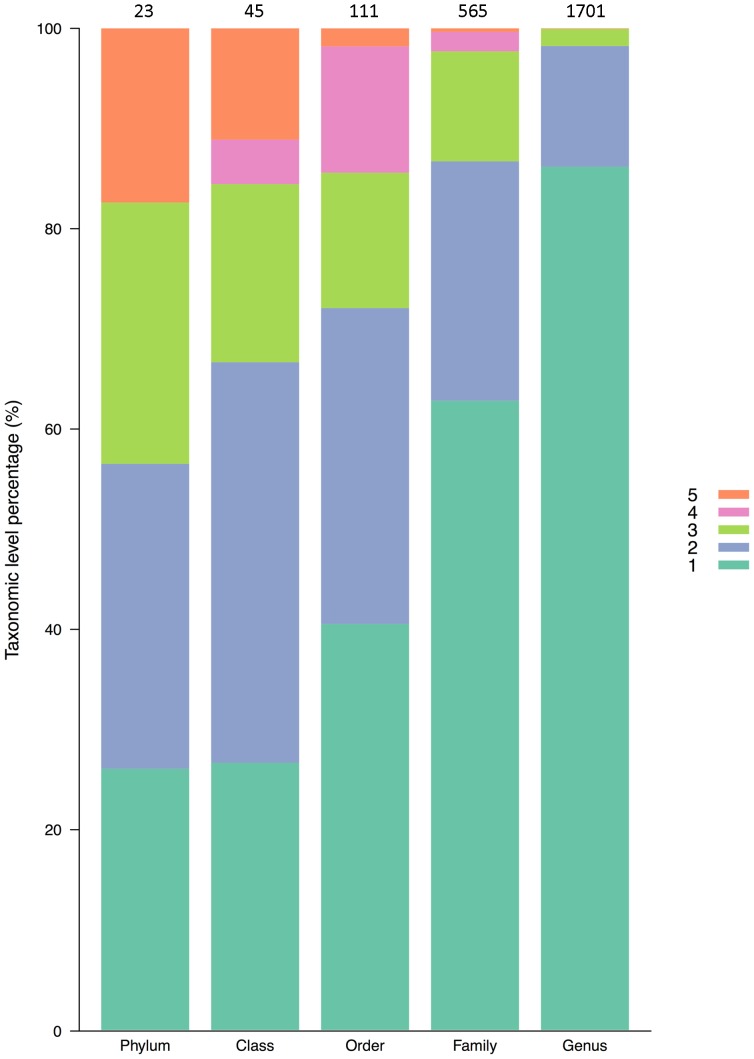
Relationship between taxonomic levels and ecological groups. Proportion of taxonomic levels composed by species belonging to the same (1) or different (2,3,4,5) ecological groups. Numbers above bars indicate the total phyla, orders, classes, families and genera and different colors indicate number of different ecological groups.

AMBI calculation requires that each identified species be assigned to an ecological group based on its taxonomic identification [12]. Because ecological groups are associated to species names, this taxonomic identification has to be as precise as to determine the species to which the individual belongs. In order to determine if taxonomic identification to higher taxonomic levels (genus, family, class or phylum) would suffice for ecological group assignment and therefore AMBI calculation, we analyzed the distribution of the AMBI species into taxonomic levels and ecological groups (Figure 1). Unfortunately, even within the same genus, there exist species belonging to different ecological groups, meaning that the identification to the species level is required for a reliable AMBI calculation.

**Figure 2:**
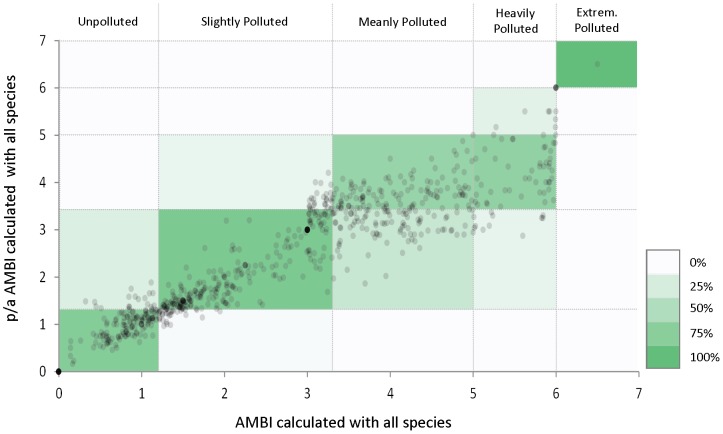
Correspondence between AMBI and p/a AMBI values. Relationship between AMBI and p/a AMBI values calculated for 694 cases. Vertical and horizontal lines indicate pollution level assessment thresholds. Color scale indicates percentage of agreement for each pollution level, meaning the number of samples that fall in the same category. Dark green color located in the diagonal reflects the best agreement between samples.

The calculation of the currently implemented AMBI is based on the number of individuals of each species found in each sample [12]. Although this information, including species abundance, could be achieved through DNA barcoding of single individuals, this method is much more time consuming and much less cost effective than metabarcoding, which consists on sequencing all individuals present in a sample at once [19]. Yet, the suitability of metabarcoding for gAMBI calculation requires further studies. Ji et al. [20] have recently shown that metabarcoding data leads to similar alpha- and beta-diversity estimates than individual taxonomic identification and, therefore, to similar policy conclusions; however, the identification of all species present in a sample with their abundances, required for the implementation of AMBI, from sequence read data in not yet possible [24]. Biological factors such as multicellularity, variation in tissue cell density, and inter and intra specific variations in gene copy number will lead to different DNA per gram of tissue extracted [40], making estimation of number of individuals from sequence data impossible. Alternatively, biomass estimations could be used to calculate BAMBI, a version of AMBI based on biomass. Though, several technical factors such as biases during DNA extraction, PCR, pooling, sequencing and bioinformatics sorting [41], [42] make estimation of biomass from sequence reads also a difficult challenge. Therefore, it seems that for now genetic data could only provide relevant information to an index that does not rely on species abundance. Fortunately, the p/a AMBI, based on presence/absence of each occurring species, provides biotic index values that are strongly related to the AMBI values [16]. This is also confirmed by our dataset from where we obtain a very good agreement (Kappa k = 0.77) between AMBI and p/a AMBI values (Figure 2). Thus, obtaining presence/absence data from genetic analyses is enough for a reliable biotic index calculation.

### AMBI species classification and available genetic data

**Figure 3:**
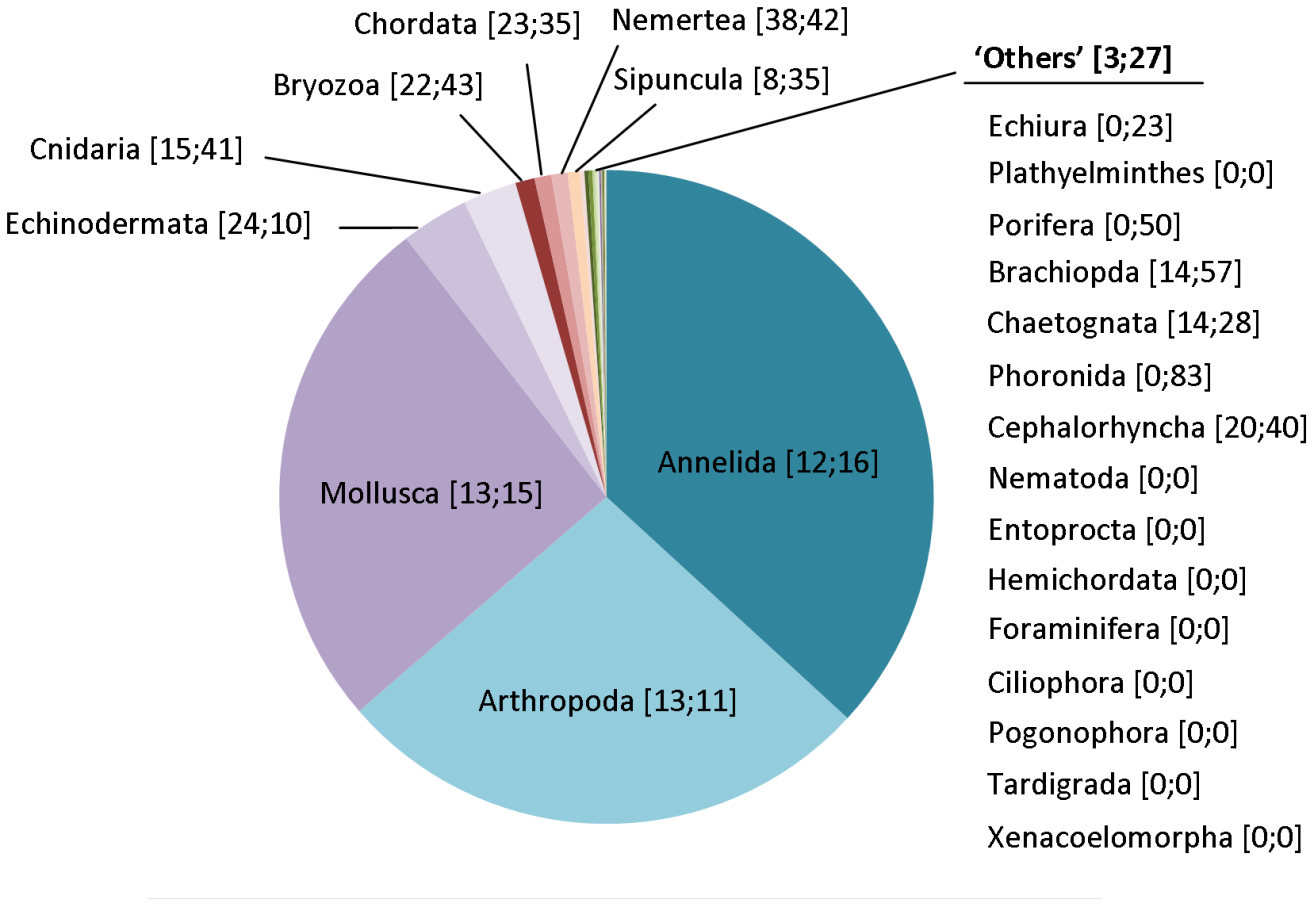
AMBI list phyla and available genetic data. Numbers in brackets indicate proportion of sequences for *CO1* or *18S rRNA* available for each phylum.

From the 5,977 taxa included in the AMBI species list, 90% fall into five phyla: Annelida (2,148 species), Mollusca (1,506 species), Arthropoda (1,448 species), Echinodermata (188 species) and Cnidaria (133 species). The remaining 10% fall into 19 phyla that contain each less than 100 taxa (Figure 3). We explored the sequences available in the GenBank database for these species for the most widely used genetic markers for animal barcoding: *CO1* and *18S rRNA* [22], [29], [30], [43]. For the former, 15,619 sequences belonging to 855 species were retrieved, whilst for the later, 2,295 sequences belonging to 940 species were retrieved. Among them, 471 species have sequences for both markers. Although the number of species for which *CO1* and *18S rRNA* sequences are available is virtually the same, more sequences for the former are available. This is due to the popularity of the *CO1* marker in barcoding studies [29] and to the extended used of this gene in molecular systematic studies leading to submission of sequences from the same species spanning different geographical areas [39], [44], [45]. Notably, only about 15% of the species included in the AMBI list have *CO1* and/or *18S rRNA* genes sequenced, which may be insufficient for the implementation of a biotic index based on barcoding or metabarcoding for taxonomic identification.

### Available sequence data is not sufficient to calculate reliable AMBI values

**Figure 4:**
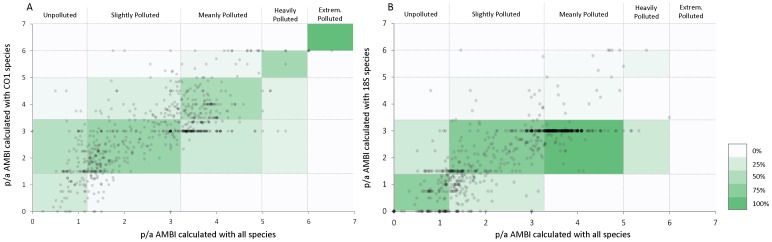
p/a AMBI values calculated with all or with only sequenced species. Relationship between p/a AMBI calculated with all species and p/a AMBI calculated with the current (A) *CO1* and (B) *18S rRNA* sequenced species. Vertical and horizontal lines indicate assessment thresholds pollution levels. Color scale as in Figure 2.

In order to determine if data from only 15% of the species in the AMBI list is sufficient to provide reliable p/a AMBI values, we gathered data from 694 cases studies (see Methods). The total number of different species found along the total serial data is 924, of which only 143 (15%) and 185 (20%) have *CO1* or/and *18S rRNA* sequenced, respectively (note that some species may have sequences for both genes). For each case study, we calculated the p/a AMBI considering all species and the p/a AMBI considering only the species with *CO1* or *18S rRNA* sequence available (Figure 4). The level of agreement between samples is fair (Kappa value of 0.502) for *CO1* and poor (Kappa value of 0.244) for *18S rRNA*, meaning that the available genetic data is not sufficient or does not fulfill the requirements for a reliable AMBI calculation.

**Figure 5:**
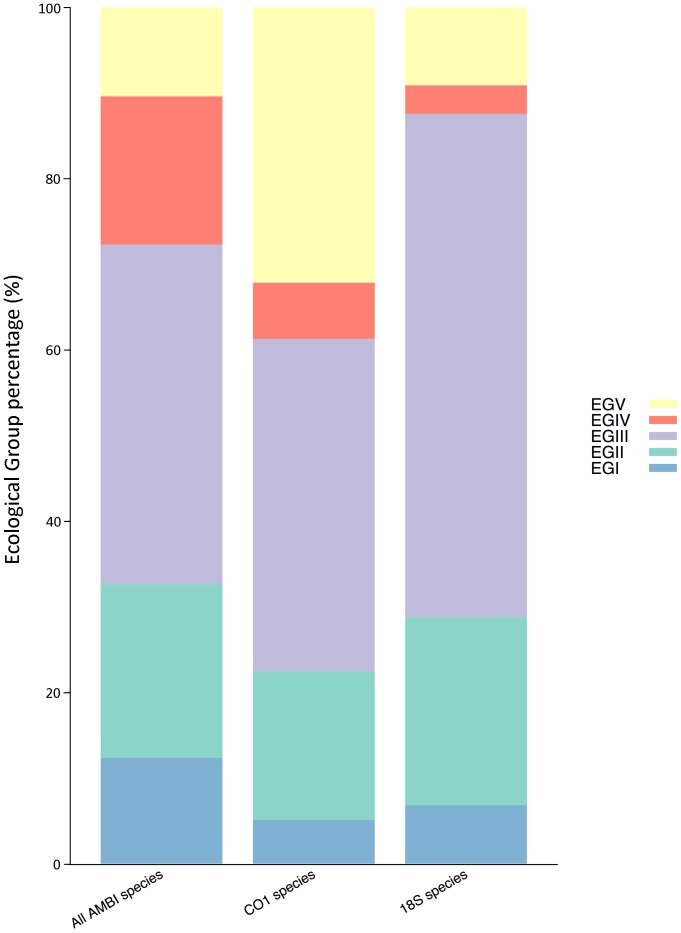
Distribution of sequenced taxa along the pollution gradient. Proportion of species, based on frequency, of each ecological group in each dataset (all species, *CO1* sequenced species and *18S rRNA* sequenced species).

Ranasinghe et al. [9] suggested that an even distribution of taxa across the disturbance gradient is needed for a reliable index calculation, condition that is not met by neither the *CO1* or *18S rRNA* datasets. Notably, the distribution of species into ecological groups of the *18S rRNA* dataset is considerably different from that of the whole dataset, being ecological group III predominant (Figure 5). This may explain the large number of cases where this dataset yields p/a AMBI of 3 regardless of the p/a AMBI values obtained with the whole dataset. Also, the slightly higher agreement obtained with the *CO1* dataset, despite being composed by less species may be explained by a more even distribution of the species into ecological groups. Thus, not only the number of species, but their distribution along the different ecological groups affects the reliability in p/a AMBI values calculation.

### How many species are necessary for an accurate AMBI calculation?

**Figure 6:**
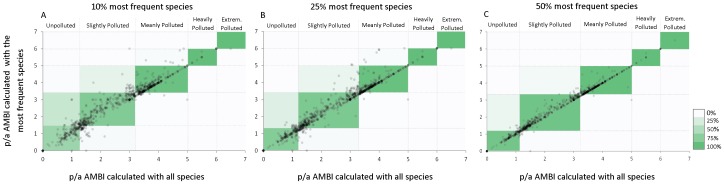
p/a AMBI calculated with all or with the most frequent species. Relationship between p/a AMBI calculated with all species and p/a AMBI calculated with the 10% (A) 25% (B) and 50% (C) most frequent species. Vertical and horizontal lines indicate assessment thresholds for pollution levels. Color scale as in Figure 2.

In order to determine the minimum number of species required to calculate accurate AMBI values, agreement tests between p/a AMBI values obtained with the full set of species and p/a AMBI values calculated with increasing percentages of the most frequent species were performed (Figure 6). Obtained Kappa values are very good (0.85 for 10% of the most frequent species) and excellent (0.93 for 25% and 0.98 for 50%). Importantly, the observed agreement is not due to the number of species selected, but to the fact that they are the most frequent ones. That is, the Kappa values obtained when using the same number of randomly selected species are significantly lower than the ones obtained using the most frequent species (p values of 1.44×10^−5^, 2.03×10^−5^ and 0.0035 for 10%, 25% and 50% respectively). Notably, the distribution of the most frequent species in ecological groups is, in all cases, similar to that of the whole species list (Figure S2). Therefore, in order to increase DNA reference library the effort must be focused on barcoding the most frequent species, which can in low number be sufficient to provide reliable p/a AMBI values.

### Evaluation of primer pairs: taxonomic coverage

Suitable genetic markers and primers that amplify the largest number of species are necessary to efficiently increase the AMBI species list reference library. We assessed the performance of primer pairs designed to amplify the most used genetic markers for Metazoa, *CO1* and *18S rRNA*, in the available sequences from these genes for the species of interest.

**Figure 7:**
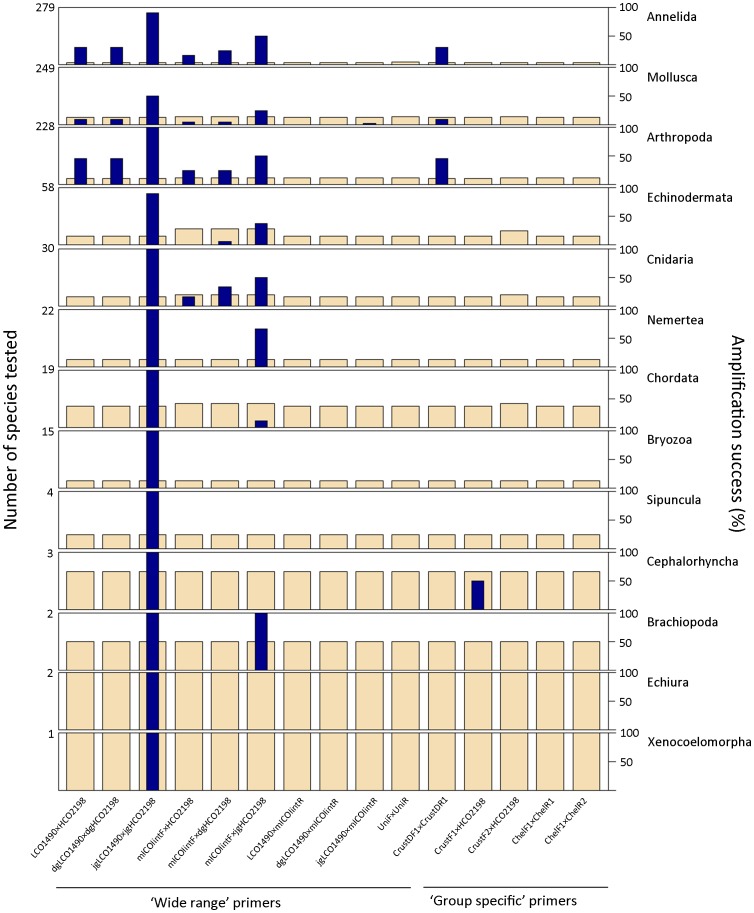
Taxa coverage for *CO1* primer pairs. Percentage of species potentially amplified for each combination of primer pair and phylum. Wheat color bars represent number of species tested per primer and dark blue color bars percentage of species (within the tested ones) potentially amplified for each primer pair. The maximum value on the left Y axis indicates the total number of species for which *CO1* sequence is available per phylum.

Despite the large number of *CO1* sequences available, very few include the complete gene sequence (Figure S1), limiting primer analysis. Thus, in order to increase the number of sequences tested in the analysis, 84 complete mitochondrial sequences - belonging to 84 species of the AMBI list - were included. Fifteen primer pairs that are included within the 658 bp ‘Folmer region’ [39], [46] were tested for 15 phyla, from which only Mollusca, Arthropoda, Echinodermata and Annelida had more than 10 sequences (Figure 7). For the remaining phyla, less than 10 sequences could be tested. Only one sequence of Hemichordata and Chaetognata was tested for each, from which no amplification was obtained with any of the primer pair (data not shown). Among the primer pairs, jgLCO1490×jgHCO2198 potentially amplify 80% of the 101 sequences tested; only Mollusca had less than 90% (50%) potentially amplifying species. Primers designed to target a shorter region (319 bp), could be tested for a higher number of species. Among them, mlCOIintF×HCO2198, mlCOIintF×dgHCO2198 and mlCOIintF×jgHCO2198 potentially amplify 9, 12 and 35%, respectively, of the 118 sequences tested.

The difference in performance of these primers could be explained by the presence of more number of degenerated bases in the last one. This could also improve the performance of the dgLCO1490×dgHCO2198 [39] pair versus the “traditional” Folmer pair, LCO1490×HCO2198, although this could not be confirmed with available sequences. Although the lack of complete sequences for *CO1* gene that include the potential primer binding sites limit our analysis, our results confirm that the degenerated primers that cover the complete Folmer region and a shorter region (319 bp) are the best performing ones [20], [26], [39], [47].

**Figure 8:**
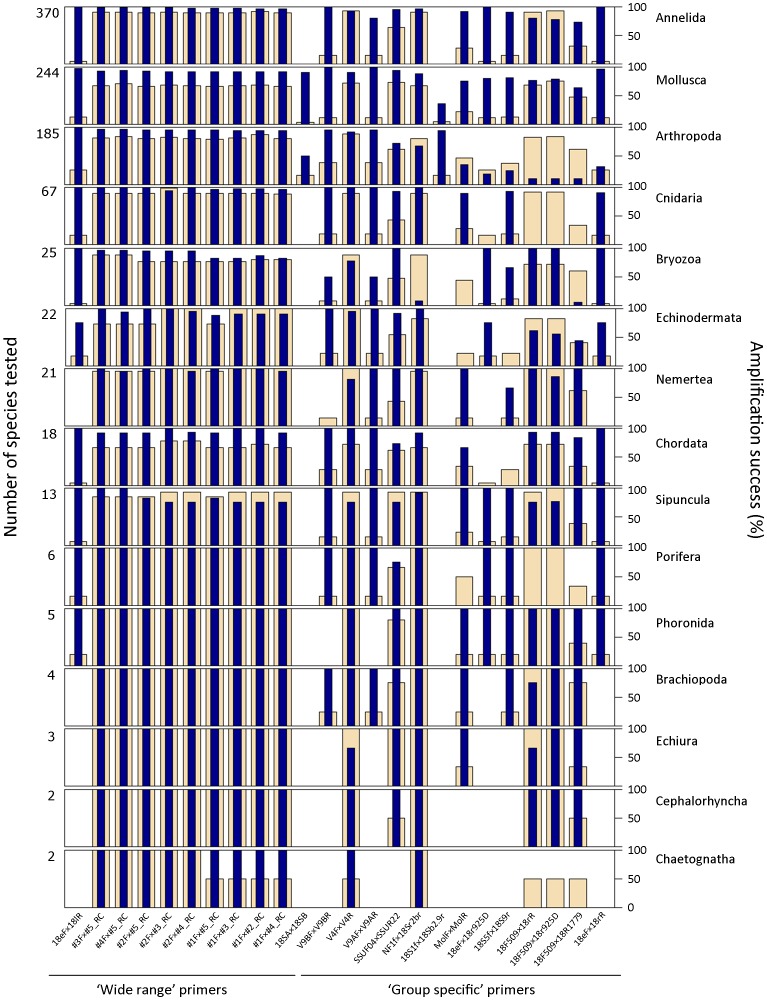
Taxa coverage for *18S rRNA* primer pairs. Percentage of species potentially amplified for each combination of primer pair and phylum. Wheat color bars represent number of species tested per primer and dark blue color bars percentage of species (within the tested ones) potentially amplified for each primer pair. The maximum value on the left Y axis indicates the total number of species for which *18S rRNA* sequence is available per phylum.

More species could be tested for *18S rRNA* data, although the reduced number of sequences available for some phyla (*e.g*. Cephalorhyncha, Chaetognata, Echinodermata, Echiura, Phoronida and Porifera) limits inferences related to these groups. The highest taxa coverage is shown for the primer pair 18eF×18lR (Figure 8), with 98% of the 118 species tested potentially amplifying; only Echinodermata and Mollusca had less than 100% (75 and 96% respectively) potentially amplifying species. Although apparently less successful in terms of percentage of species potentially amplifying among the tested ones (ranging from 97.1 to 94.2%), the remaining universal primers could be tested in all phyla. In particular, primer pair #3Fx#5_RC has an amplification success of 97.1% and all phyla and almost all species could be tested. Thus, according to our results, primer pair #3Fx#5_RC is the best performing for *18S rRNA* macroinvertebrate amplification. The primer pair selected by other authors as best performing [32] also provides successful amplification rates although slightly lower (94%).

### DNA barcoding of AMBI species

In order to start increasing the reference library for a future gAMBI, we attempted to sequence the *CO1* gene fragment amplified with the dgLCO1490×dgHCO2198 primer pair from the most frequent species. From 115 individuals selected, 56 amplified and 22 gave a sequencing product. The specimens have been submitted to BOLD (http://www.boldsystems.org) with BINs BOLD:AAJ1248, ACJ4563, ACJ4767, ACH4094, ACJ2906, ACG2010, ACJ4318, ACJ2494, ABU8508, ACJ4125, ACJ4592, ACJ4543, ABA9346, ACJ2932, ACJ2637, ACJ2931, ACJ4785, ACJ4313, ACJ2499, ACJ2492, ACJ2498 and ACJ4512; and the sequences deposited in GenBank with accession numbers KF808157 - KF808178. The 22 new sequenced species have been included in the list of sequenced *CO1* species for p/a AMBI calculations. Among them, 8 taxa (*Magelona johnstoni, Urothoe pulchella, Protodorvillea kefersteini, Polygordius appendiculatus, Glycera unicornis, Diogenes pugilator*, *Scolaricia* sp. and *Glycinde nordmanni*) are within the 10% most frequent, 6 (*Ampelisca sarsi*, *Chamelea striatula*, *Phyllodoce lineata*, *Pseudomystides limbata*, *Necallianassa truncata* and *Haplostylus normani*), within the 25% most frequent and 4 (*Hyala vitrea, Sabellaria spinulosa, Bathyporeia tenuipes* and *Paradoneis ilvana*), within the 50% most frequent taxa, whilst 4 taxa (*Thracia phaseolina, Paradoneis sp., Magelona minuta* and *Sthenelais limicola*) are not part of the most frequent species. The level of agreement between p/a AMBI calculated with all species and p/a AMBI calculated with *CO1* species (included the abovementioned) is good (Kappa value of 0.617), improving the one obtained with the previously available resources for this gene.

## Outlook

Overall, our results place DNA barcoding as a viable alternative to visual species identification in the context of taxonomic assignment for gAMBI calculation; though, this viability is subject to increasing the number of sequences in the reference library. According to our results, this increase should be performed focusing on the most frequently occurring species, as their presence in the reference library, even in a small percentage, is enough for an accurate gAMBI calculation.

Here, we have focused on the use of (meta) barcoding techniques to ease the first step for the calculation of AMBI: taxonomic identification. However, it could be possible to think about a new version of gAMBI based on total biodiversity metabarcoding profile that would not require finding a particular set of species previously defined. Therefore, besides working on increasing the gAMBI reference library, we are also focusing on comparing samples analyzed by visual taxonomy and by metabarcoding in order to explore more practical genetics based alternatives to AMBI.

Regardless of whether we pursue species or higher taxonomic level identification, increasing the reference library of sequences is mandatory, and even if the cost of doing so depends on many factors, there is no doubt that it will remain significant [19]. Yet, once the initial investment for building the library is made, each individual in a sample can be identified by DNA barcoding per about $5 [48], and a whole sample per about $50 if it is bulk processed by metabarcoding (rough calculation assuming multiplexing 100 samples on the Illumina MiSeq platform and without considering the bioinformatics processing of the data). Needing still optimization of several analytical steps, the optimal cost-efficiency of DNA techniques for taxonomic identification has not yet been achieved, but has already overtaken that of visual identification [49].

Our ultimate goal is to develop genetics based tools for a cheaper and faster assessment of the marine quality, which is nowadays suffering from methodological and budget limitations [17]. Besides their cost-efficiency, genomics based methods allow a rapid and reliable identification of specimens, irrespective of the taxonomic group or available taxonomic expertise. Showing that a genomics based AMBI is a viable alternative to a morphological identification based AMBI, we foresee the use of this index for monitoring regions where no taxonomic expertise and/or sufficient monitoring budget is available.

## Supporting Information

Figure S1 Primer pair positions. Position of the primer pairs tested for *CO1* (A) on the *CO1* region of the complete mitochondrial gene of *Mytilus galloprovincialis* (Accession number DQ399833) and for *18S rRNA* (B) on the *18S rRNA* sequence of *Aplysia punctata* (Accession number AJ224919).
(TIF)

Figure S2 Distribution of most frequent taxa along the pollution gradient. Proportion of species, based on frequency, of each ecological group in each dataset (all species, 10% most frequent, 25% most frequent and 50% most frequent).
(TIF)

Table S1 Primer pairs tested for CO1 and 18S rRNA sequences available in public databases.
(PDF)
