# Supplementary figures and images for: Environmental Status Assessment Using DNA Metabarcoding: Towards a Genetics Based Marine Biotic Index (gAMBI)
Source: PLoS One. 2014 Mar 6;9(3):e90529. doi: 10.1371/journal.pone.0090529 (PMC3946187; doi:10.1371/journal.pone.0090529)

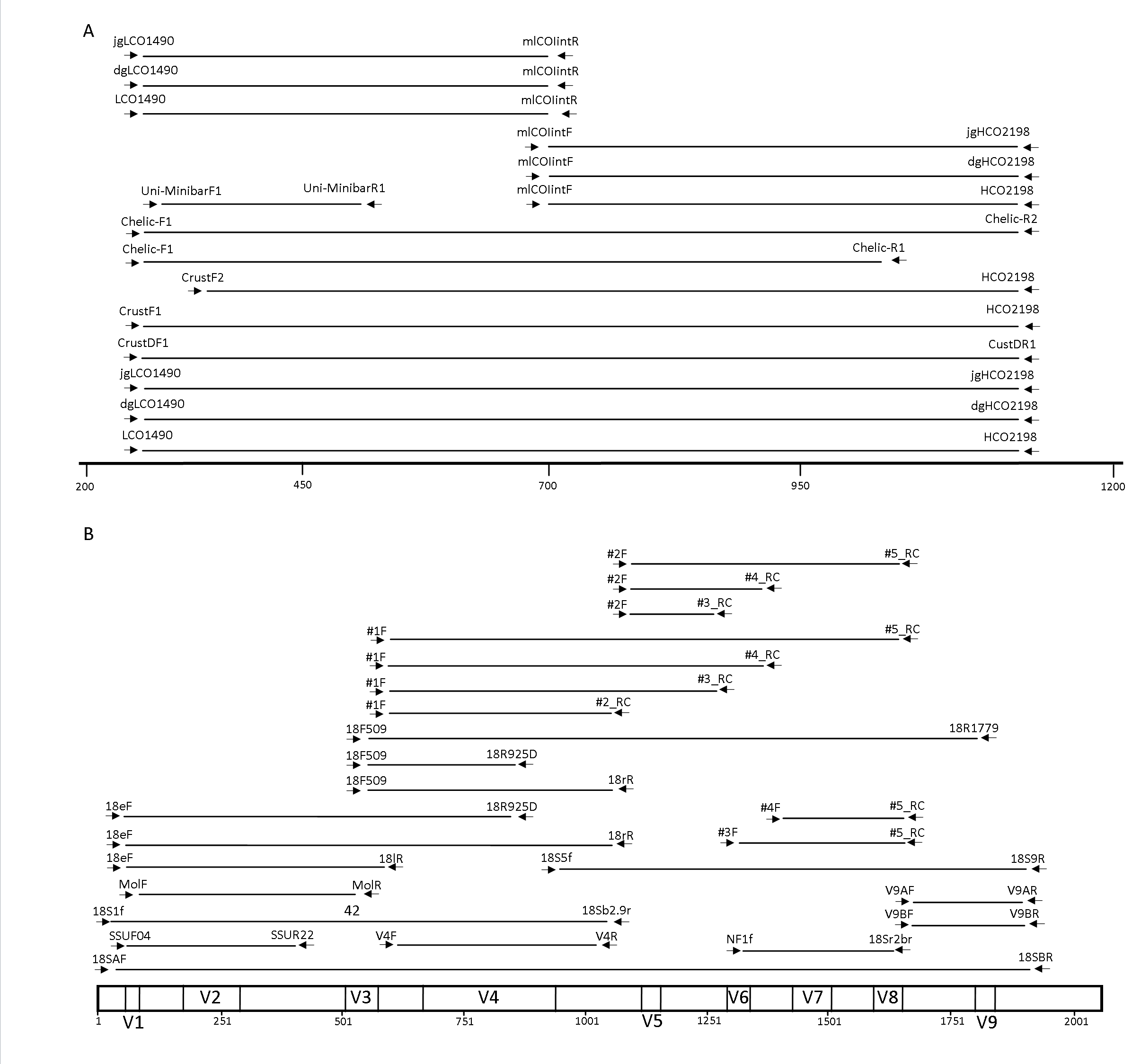

Supplement: Figure S1 — Primer pair positions. Position of the primer pairs tested for CO1 (A) on the CO1 region of the complete mitochondrial gene of Mytilus galloprovincialis (Accession number DQ399833) and for 18S rRNA (B) on the 18S rRNA sequence of Aplysia punctata (Accession number AJ224919). (TIF) [file pone.0090529.s001.tif]

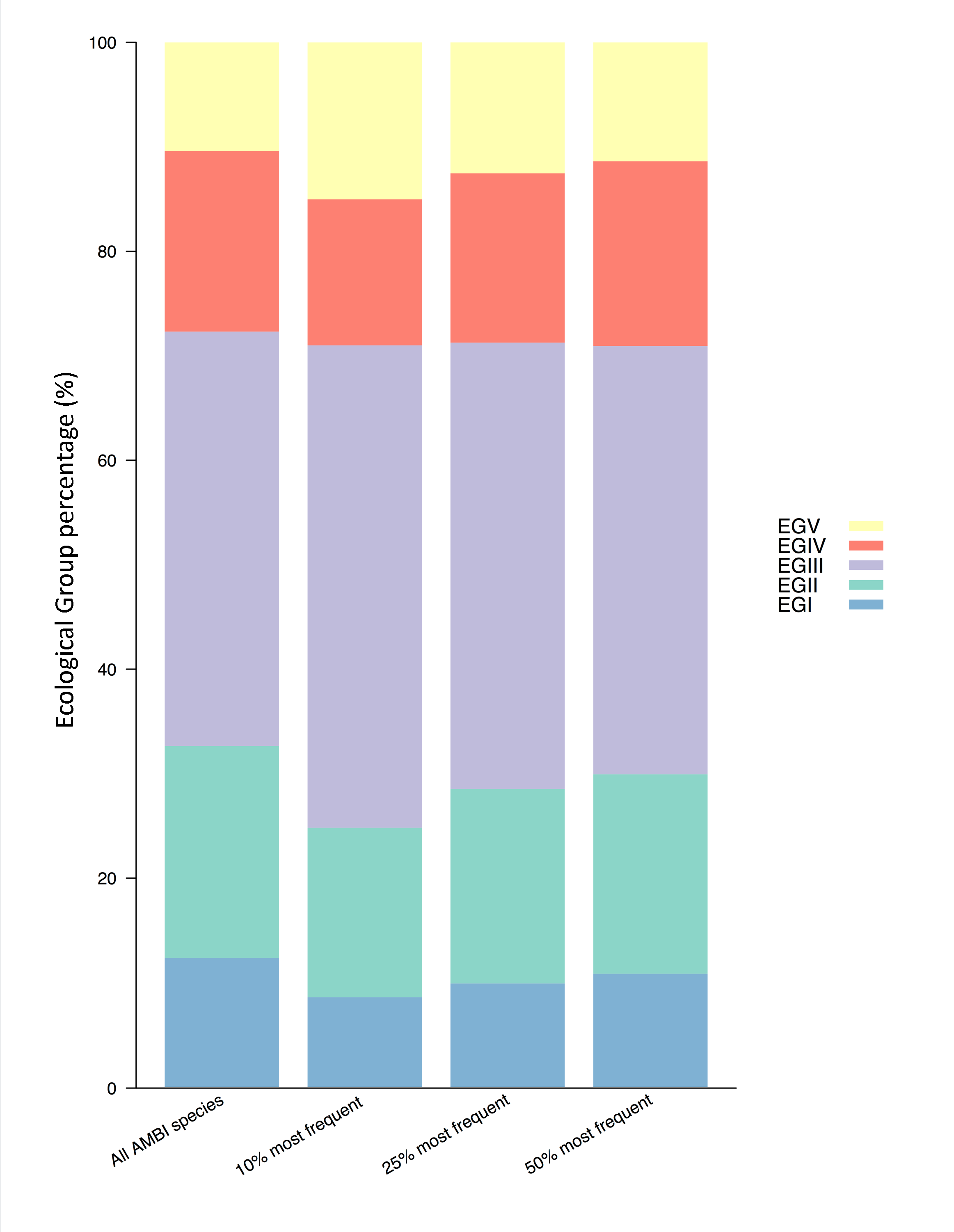

Supplement: Figure S2 — Distribution of most frequent taxa along the pollution gradient. Proportion of species, based on frequency, of each ecological group in each dataset (all species, 10% most frequent, 25% most frequent and 50% most frequent). (TIF) [file pone.0090529.s002.tif]
