# Supplementary material for: Environmental Status Assessment Using DNA Metabarcoding: Towards a Genetics Based Marine Biotic Index (gAMBI)
Source: PLoS One. 2014 Mar 6;9(3):e90529. doi: 10.1371/journal.pone.0090529 (PMC3946187; doi:10.1371/journal.pone.0090529)
Supplement: Table S1 — Primer pairs tested for CO1 and 18S rRNA sequences available in public databases. (PDF) [file pone.0090529.s003.pdf]

Table S1. Primer pairs tested for CO1 and 18S rRNA sequences available in public databases.

**Primer pair**	**5’ – 3’ Forward primer sequence**	**5’ – 3’ Reverse primer sequence**	**Reference**
**CO1**			
LCO1490 × HC02198	GGTCAACAAATCATAAAGATATTGG	TAAACTTCAGGGTGACCAAAAAATCA	[1]
dgLCO × dgHCO	GGTCAACAAATCATAAAGAYATYGG	TAAACTTCAGGGTGACCAAARAAYCA	[2]
jgLCO × jgHCO	TITCIACIAAYCAYAARGAYATTGG	TAIACYTCIGGRTGICCRAARAAYCA	[3]
LCO1490 × mlCOIintR	See above	GGRGGRTASACSGTTCASCCSGTSCC	[4]
dgLCO × mlCOIintR	See above	See above	[4]
jgLCO × mlCOIintR	See above	See above	[4]
mlCOIintF × HC02198	GGWACWGGWTGAACWGTWTAYCCYCC	See above	[4]
mlCOIintF × dgHCO	See above	See above	[4]
mlCOIintF × jgHCO	See above	See above	[4]
UniF × UniR	CAAAATCATAATGAAGGCATGAGC	TCCACTAATCACAARGATATTGGTAC	[5]
CrustDF1 × CrustDR1	GGTCWACAAAYCATAAAGAYATTGG	TAAACYTCAGGRTGACCRAARAAYCA	[6]
CrustF1 × HC02198	TTTTCTACAAATCATAAAGACATTGG	See above	[7]
CrustF2 × HC02198	GGTTCTTCTCCACCAACCACAARGAYAT HGG	See above	[7]
ChelF1 × ChelR1	TACTCTACTAATCATAAAGACATTGG	CCTCCTCCTGAAGGGTCAAAAAATGA	[8]
ChelF1 × ChelR2	See above	GGATGGCCAAAAAATCAAAATAAATG	[8]
**18S**			
18SA × 18SB	AACCTGGTTGATCCTGCCAGT	TGATCCTTCCGCAGGTTCACCT	[9]
SSUF04 × SSUR22	GCTTGTAAAGATTAAGCC	GCCTGCTGCCTTCCTTGGA	[10]
NF1f × 18Sr2br	GGTGGTGCATGGCCGTTCTTAGTT	TACAAAGGGCAGGGACGTAAT	[11]
V4F × V4R	CCAGCASCYGCGGTAATTCC	ACTTTCGTTCTTGATYRA	[12]
V9AF × V9AR	GTACACACCGCCCGTC	TGATCCTTCTGCAGGTTCACCTAC	[12]
V9BF × V9BR	TTGTACACACCGCCC	CCTTCYGCAGGTTCACCTAC	[13]
18S1f × 18Sb2.9r	TACCTGGTTGATCCTGCCAGTAG	TATCTGATCGCCTTCGAACCTCT	[14]
18S5f × 18S9r	GCGAAAGCATTTGCCAAGAA	GATCCTTCCGCAGGTTCACCTAC	[15]
MolF × MolR	GCCAGTAGCATATGCTTGTCTC	AGACTTGCCTCCAATGGATCC	[16]
18eF × 18lR	CTGGTTGATCCTGCCAGT	GAATTACCGCGGCTGCTGGCACC	[17,18]
18eF × 18R925D	See above	GATCYAAGAATTTCACCTCT	[17,19]
18eF × 18rR	See above	GTCCCCTTCCGTCAATTYCTTTAAG	[17,20]
18F509 × 18rR	CCCCGTAATTGGAATGAGTACA	See above	[20,21]
18F509 × 18R925D	See above	See above	[19,21]
18F509 × 18R1779	See above	TGTTACGACTTTTACTTCCTCTA	[21]
#1F × #2_RC	CTGGTGCCAGCAGCCGCGGYAA	TCCGTCAATTYCTTTAAGTT	[22]
#1F × #3_RC	See above	AACYAMSAACGGCCATGCACCRC	[22]
#1F × #4_RC	See above	CKRAGGGCATYACWGACCTGTTAT	[22]
#1F × #5_RC	See above	GTGTGYACAAAGGBCAGGGAC	[22]
#2F × #3_RC	AACTTAAAGRAATTGACGGA	See above	[22]
#2F × #4_RC	See above	See above	[22]
#2F × #5_RC	See above	See above	[22]
#3F × #5_RC	GYGGTGCATGGCCGTTSKTRGTT	See above	[22]
#4F × #5_RC	ATAACAGGTCWGTRATGCCCTYMG	See above	[22]

References

1. Folmer O, Black M, Hoeh W, Lutz R, Vrijenhoek R (1994) DNA primers for amplification of mitochondrial cytochrome c oxidase subunit I from diverse metazoan invertebrates. Molecular Marine Biology and Biotechnology 3: 294-299.
2. Meyer CP (2003) Molecular systematics of cowries (Gastropoda: Cypraeidae) and diversification patterns in the tropics. Biological Journal of the Linnean Society 79: 60.
3. Geller J, Meyer C, Parker M, Hawk H (2013) Redesign of PCR primers for mitochondrial cytochrome c oxidase subunit I for marine invertebrates and application in all-taxa biotic surveys. Mol Ecol Resour 13: 851-861.
4. Leray M, Yang YJ, Meyer PC, Mills CS, Agudelo N, et al. (2013) A new versatile primer set targeting a short fragment of the mitochondrial COI region for metabarcoding metazoan diversity: application for characterizing coral reef fish gut contents. Front Zool 10.
5. Meusnier I, Singer G, Landry J, Hickey D, Hebert P, et al. (2008) A universal DNA mini-barcode for biodiversity analysis. Bmc Genomics 9.
6. Radulovici AE, Sainte-Marie B, Dufresne F (2009) DNA barcoding of marine crustaceans from the estuary and gulf of st lawrence: A regional-scale approach. Molecular ecology resources 9: 181-187.
7. Costa FO, deWaard JR, Boutillier J, Ratnasingham S, Dooh RT, et al. (2007) Biological identifications through DNA barcodes: the case of the Crustacea. Canadian Journal of Fisheries and Aquatic Sciences 64: 272-295.
8. Barrett RDH, Hebert PDN (2005) Identifying spiders through DNA barcodes. Canadian Journal of Zoology 83: 481-491.
9. Apakupakul K, Siddall ME, Burreson EM (1998) Higher Level Relationships of Leeches (Annelida: Clitellata: Euhirudinea) Based on Morphology and Gene Sequences. Molecular Phylogenetics and Evolution 12: 10.
10. Blaxter ML, De Ley P, Garey JR (1998) A molecular evolutionary framework for the phylum Nematoda. Nature 392.
11. Porazinska DL, Giblin-Davis RM, Faller L, Farmerie W, Kanzaki N, et al. (2009) Evaluating high-throughput sequencing as a method for metagenomic analysis of nematode diversity. Mol Ecol Resour 9: 1439-1450.
12. Guillou L, Bachar D, Audic S, Bass D, Berney C, et al. (2012) The Protist Ribosomal Reference database (PR2): a catalog of unicellular eukaryote small sub-unit rRNA sequences with curated taxonomy. Nucleic Acids Res 41: D597-604.
13. Amaral-Zettle LA, McCliment EA, Ducklow HW, Huse SM (2009) A Method for Studying Protistan Diversity Using Massively Parallel Sequencing of V9 Hypervariable Regions of Small-Subunit Ribosomal RNA Genes. PLoS ONE.
14. Whiting MF (2002) Mecoptera is paraphyletic: multiple genes and phylogeny of Mecoptera and Siphonaptera. Zoologica Scripta 31.
15. Carranza S, Giribet G, Ribera C, Baguña J, Riutort M (1996) Evidence that two types of 18S rDNA coexist in the genome of Dugesia (Schmidtea) mediterranea (Platyhelminthes, Turbellaria, Tricladida). Molecular Biology and Evolution 13.
16. Holland PWH, Hacker AM, Williams NA (1991) A molecular analysis of the phylogenetic affinities of *Saccoglossus cambrensis* brambell & cole (hemichordata). Phil Trans R Soc Land B 332: 185-189.
17. Hillis DM, Dixon MT (1991) Ribosomal DNA: Molecular evolution and phylogenetic inference. . Quarterly Review of Biology 53: 66:411.
18. Halanych K, Bacheller J, Aguinaldo A, Liva S, Hilli D, et al. (1995) Evidence from 18S ribosomal DNA that the lophophorates are protostome animals. Science 3.
19. Burnette A, Struck TH, Halanych KM (2005) Holopelagic Poeobius meseres (Poeobiidae, Annelida) is derived from within benthic Flabelligerid worms. Biological Bulletin 20: 208:213.
20. Passamaneck YJ, Schander C, Halanych KM (2004) Investigation of molluscan phylogeny using large-subunit and small-subunit nuclear rRNA sequences. Molecular Phylogenetics and Evolution 38: 32:25.
21. Struck TH, Purschke G, Halanych KM (2005) A scaleless scale worm: Molecular evidence for the phylogenetic placement of *Pisione remota*(Pisionidae, Annelida). Marine Biology Research 1: 243-253.
22. Machida JR, Knowlton N (2012) PCR Primers for Metazoan Nuclear 18S and 28S Ribosomal DNA Sequences. PLoS ONE 7.
